# A case control study investigating the effects of levels of physical activity at work as a risk factor for prostate cancer

**DOI:** 10.1186/1476-069X-13-64

**Published:** 2014-08-07

**Authors:** Glenn W Doolan, Geza Benke, Graham G Giles, Gianluca Severi, Timo Kauppinen

**Affiliations:** 1 Department of Epidemiology & Preventive Medicine, Monash University, The Alfred Centre, The Alfred, Commercial Road, Melbourne, Victoria 3004, Australia; 2 Cancer Epidemiology Centre, Cancer Council Victoria, 615 St. Kilda Road, Melbourne, Victoria 3004, Australia; 3 Centre for Genetic Epidemiology, University of Melbourne, 200 Berkeley Street, Carlton, Victoria 3053, Australia; 4 Finnish Institute of Occupational Health, Topeliuksenkatu 41aA, FIN-00250 Helsinki, Finland; 5 Permanent Address: P.O. Box 276, Trafalgar, Victoria 3824, Australia

**Keywords:** Manual handling of burdens, Occupational exposure, Physical activity, Physical workloads, Prostate cancer, Risk factors, Finish job exposure matrix

## Abstract

**Background:**

A potential risk factor for prostate cancer is occupational physical activity. The occupational aetiology of prostate cancer remains unclear. The purpose of this research was to examine associations between the level of exposure to various measures of physical activity at work and the risk of Prostate Cancer.

**Methods:**

Using the Finnish Job Exposure Matrix and the occupational history of 1,436 cases and 1,349 matched controls from an Australian case control study; we investigated five related exposure variables considered to be risk factors by comparing odds ratios.

**Results:**

Modestly increasing odds ratios were detected with increasing levels of *workload* but there was no difference in this trend between moderate and high grade tumours. In regard to occupational *physical workload* no statistically significant association was observed overall but an increasing trend with level of exposure was observed for high grade compared with moderate grade tumours.

**Conclusion:**

Both *workload* and *physical workload* merit further investigation, particularly for the latter in relation to grade of tumour.

## Background

Many past studies have investigated various occupational chemical and physical agents as likely causes of prostate cancer [1]. When investigating the causes of death after the diagnosis of prostate cancer it has also been previously found that men with low to moderate grade prostate cancer had a similar rate of death to men without prostate cancer [2]. There are very few well established risk factors of prostate cancer especially those that are potentially modifiable risk factors [3]. Therefore the rationale for this study is to investigate the likely association of some modifiable occupational risk factors and prostate cancer. Previous reported studies investigating the role that physical activity plays in the occupational environment, have described physical activity by various metrics [4,5]. Ricciardi provided a model for the concept of *Sedentarism* that included attributes such as expending less than 10% daily energy in the performance of moderate and high-intensity activities in which the metabolic rate increases at least four times from baseline, or not engaging in physical activities five or more times per week or no leisure activity or no physical activity for up to 3hrs per week that increases the metabolic rate by four times from base [4]. In relation to Leisure Time Physical Activity (LTPA), Kirk found that those employed in occupations demanding long work hours and low Occupational Physical Activity (OPA) are at higher risk of inactivity.

Some authors [6] have demonstrated that men who participated in regular LTPA reduced their risk for clinical prostate cancer and in the workplace concluded that physical activity at work was also beneficial in reducing the risk of prostate cancer [7-10]. However, Bairati et al. suggested that socio-economic status was a probable confounder [10]. In two systematic reviews, it was found that few studies demonstrate a protective role of OPA, even though high levels of OPA and LTPA together seemed to reduce the risk of advanced prostate cancers [11,12]. A further review concluded there was inconsistent evidence for an inverse association between OPA and prostate cancer [13]. Recently, a meta-analysis [14] found no clear evidence for an association between job strain and the risk of prostate cancer in relation to OPA. Discacciati et al., adds another dimension to the overall picture by concluding that obesity may have a dual effect on PCa by a decreased risk of low grade PCa and an increased risk of high grade PCa [15].

In contra distinction, a positive association was reported [3] between prostate cancer risk and the highest category of workplace physical activity, which is the opposite of what has been reported by most other studies [7-10] of physical activity and prostate cancer.

Our aim was to investigate whether an association existed between occupational physical activity exposures (assessed using FINJEM ) and prostate cancer, and, in order to address issues of possible detection bias, also to inspect whether such associations differed by grade of tumour. Occupational studies using job exposure matrices (JEMs) have reported some associations with prostate cancer risk, but these have not consistently been replicated by other studies [16]. Although leisure time physical activity may be a limitation and potential cause of bias due to misclassification, there is no reason to suggest that the profile of the cases are different to the controls. This article specifically discusses the reported OPA in men in relation to prostate cancer using the demographic profile of the sample and the odds ratios found in relation to five exposure variables (that are possible risk factors) measuring different forms of workplace physical activity, rather than LTPA. In this study we have used the term ‘occupational exposure’ to include both ergonomic and psychological variables.

## Methods

Giles et al. [17] has reported on the Australian Prostate Cancer study elsewhere. Briefly, population based cancer registries in Melbourne, Sydney and Perth were utilized to recruit a random sample of 2,528 cases with prostate cancer diagnosed at age 39–80 and 3,125 controls which were considered eligible at the time of selection. For the purposes of this analysis the number of participants was reduced due to factors such as no access to patient records, refusal of controls, insufficient English skills, or moved address. Further analysis was restricted to 1,495 (65%) cases with prostate cancer diagnosed at age 39–70 and 1423 (46%) controls aged between 40 and 70 years. The final analysis for which there was sufficient information regarding occupational work histories included 1,436 cases (96%) and 1,349 (94%) controls, aged between 39 and 70 years.

Participants were also asked to complete a Lifetime Calender of residence and employment in order to prompt more complete answers when responding to the study questionnaires. The controls were matched through frequency based matching with age and were free of prostate cancer upon recruitment. Recruitment was stratified by age and all men under the age of 60 years were invited to participate. Initially, random samples of 50% of men aged 60–64 and 25% of men aged 65–70 were selected, with the proportions varying overtime to fit interview quotas.

Cases recruited in Melbourne, Sydney and Perth, Australia were diagnosed in the study period and notified to the population-based cancer registries with a histopathologically-confirmed diagnosis of adenocarcenoma of the prostate, and excluded tumours that were well-differentiated (defined as low grade tumours, that is, those with a Gleason score of less than five).

A major concern with prostate cancer is the diagnostic staging and whether any occupational exposures are associated with medium or high grade cancers. One approach to overcoming concerns regarding the inclusion of clinically unimportant tumours as cases is to select cases who are diagnosed in the study period and notified to the population-based cancer registries with a histopathologically confirmed diagnosis of adenocarcenoma of the prostate, excluding tumours that are well-differentiated (defined as low grade tumours i.e. those with a Gleason score less than five). This has been addressed in this study.

The self-reported data from the calendar and questionnaires that related to occupation were collated together with date-of-birth, location, children and their gender, and school/occupation and linked with other clinical data variables from study such as smoking, alcohol consumption and physical activity at work. This data was has been shown to be both valid and reliable in other analysis [17].

This further analysis of Giles et al. original study [17] was undertaken using de-identified data and is covered by the AVCC Institutional Ethics Committee permission (1992) from the Anti-Cancer Council of Victoria, and permission from the Chief Investigator of the *Risk Factors for Prostate Cancer Case–control Study (2004).*

### Exposure assessment

For exposure assessment we used FINJEM [18], a community-based job exposure matrix, originally developed by the Finnish Institute of Occupational Health, for use in epidemiological studies. FINJEM covers a wide range of physical, chemical, microbiological, ergonomic and psychological exposures and is the only job exposure matrix that covers all the different types of radiation. FINJEM is coded for 311 classes of occupation, according to the Finnish occupation coding classification. The exposure is measured in line with the method described by Kauppinen at al. [19]. Each job or employment episode of the participants in this study was coded according to the Finnish occupation coding classification and a FINJEM code number (O-Code) was allocated. The coding facilitated the linkage between the occupational activity exposures and prostate cancer status. As an example of this linkage please see Table 1 for a list of the three top occupations with the highest levels of exposures for each exposure variable. It is noted that this list should be treated cautiously as quite a few men had more than one occupation during the course of their working life.

**Table 1:** A list of top three occupations with the highest levels of exposures for each exposure variable for men with high grade tumours

**Manual handling of burdens**	**Sedentary work**	**Physical workload**	**Total cumulative activity-over-time**	**Work load**
Cabinet maker	Taxi driver	Meat worker	Meat worker	Editor
Meat worker	Truck driver	Cabinet maker	Sailor	Travel consultant
Market gardener	Draftsman	Cleaner	Policeman	Ships officer

Each exposure variable has a specific definition and value and exposure is characterised by the proportion of exposed workers *(P)* and the mean level of exposure *(L)* and is given as *P × L* for each occupation. Cumulative exposure was calculated by P × L × Years exposed in the various exposed jobs reported by the participant. Occupational exposures that did not exceed the non-occupational background level were omitted for example, background radiation levels).

The cumulative exposure for the exposed participants was calculated in tertiles and quartiles. In the model used it included occupational exposure variable plus age, family history and the SEIFA index of economic resources, which is a measure of socio-economic disadvantage, first produced by the Australian Bureau of Statistics [20] following the 1971 census. Only occupational cumulative exposures from our model are presented in the results as it takes into account the level of economic and social disadvantage within the sample, as well as age and family history confounders.

### The exposure variables

We investigated three exposure variables; manual handling of burdens, physical workloads, sedentary work, and two created variables of cumulative activity-over-time and workload by comparing odds ratios in tertiles and quartiles through analysis by binary logistic regression. Moderate and high grade tumours were compared using polytomous regression. The *manual handling of burdens* consists of lifting, and carrying of heavy burdens, and is an essential feature of the everyday work tasks. *Physical workloads* consist of tasks where the whole body is exerted by dynamic muscular work. *Sedentary work* consists of work done in seated posture [19]. Also, one of the variables were created, total cumulative activity-over-time is calculated by adding an individual’s total scores over their disclosed working life so that some comparison could be undertaken in regard to working in high to low activity jobs for a prolonged period. The psychological exposure variable *Workload* is a measurement of the overall psychological impact of perceived occupational load over the years of employment. If a subject considers they have been stressed from a high workload over the majority of their working history, it might indicate that, as a stressor, this could have a long-term harmful outcome. *Workload* is defined as a psychological factor in FINJEM [19] and is derived from the demand to work under tight schedules and time pressure, and to adjust conflicting demands from others subjective perceptions.

## Results

The profile of the sample in Table 2 shows the ages of the participants were relatively evenly spread between cases and controls. In the 65–70 year age group, this group was slightly larger and consistent with the expected occurrence and diagnosis with the control group having the greater number of participants below the age of 55 years.

**Table 2:** Demographic description of characteristics of cases and controls

**Variable**	***Cases (n = 1436)***		***No. of controls (n = 1349)***	
**Age group**	*n*	*%*	*n*	*%*
39- 55	296	20.6	329	24.4
55 - 59	328	22.8	217	16.1
60 - 64	359	25.0	398	29.5
65 - 70	453	31.5	405	30.0
**Country of birth**				
Australia	988	68.8	816	60.5
Not-Australia	448	31.2	533	39.5
**Educational level**				
Primary only	97	6.8	135	10.0
Secondary only	459	32.1	426	31.7
Post-secondary training	633	44.2	581	43.2
Tertiary	243	17.0	202	15.0
**Family history**				
No first degree relative affected	1180	82.6	1257	93.8
At least one first degree relative affected	249	17.4	83	6.2
**Marital status**				
Married/de facto	1230	85.8	1140	85.2
Once married	159	11.1	142	10.8
Never married	45	3.1	65	4.0
**State**				
New South Wales	419	29.2	319	23.6
Victoria	767	53.4	781	57.9
Western Australia	250	17.4	249	18.5

There were ten percent more Australian born cases than controls. Educationally both groups were closely matched, but numerically the control group had a higher number of men with lower educational attainment. In regard to family history, cases had an 11.2% greater difference of at least one first degree relative being affected by prostate cancer. Marital status had a similar spread in both groups.

Table 3 shows the results of the binary logistic regression for the five exposure variables, manual handling of burdens, sedentary work, workload, cumulative activity-over-time and physical workloads. None of the three ergonomic factors, manual handling of burdens, physical workload and sedentary work were associated with prostate cancer risk, nor was the calculated variable of cumulative activity-over-time. The psychological variable of *workload* which measures the worker’s perceptions in relation to an occupational lifetime of high workload activity was the only statistically significant association and it showed a positive relationship with PCa risk.

**Table 3:** Cumulative occupational exposures for prostate cancer by binary logistic regression

**Binary logistic regression**								
**Exposure total**	**Exposure**	**Cumulative exposure**		**Controls**	**Cases**	OR*	**95% CI**	**p for trend (unadjusted model)**
**Manual handling of burdens (score)**	1^st^ Tertile	0	- ≤ 2.654	472	534	1.00		
	2^nd^ Tertile	> 2.655	- ≤ 6.808	440	443	0.96	0.79 – 1.15	
	3^rd^ Tertile	> 6.808		437	459	1.01	0.84 – 1.22	
	Continuous			1349	1436			0.82
**Sedentary work (score)**	Unexposed	0		843	912	1.00		
	1^st^ Tertile	>0	– ≤ 1.6	158	177	0.96	0.78 – 1.25	
	2^nd^ Tertile	>1.6	- ≤ 5.108	180	161	0.78	0.62- 0.99	
	3^rd^ Tertile	>5.108		168	186	1.02	0.81- 1.29	
	Continuous			1349	1436			0.94
**Work load (score)**	1^st^ Tertile	> 0	- ≤ 102	426	366	1.00		
	2^nd^ Tertile	> 103	- ≤ 127	466	496	1.20	0.99 – 1.46	
	3^rd^ Tertile	> 128		457	574	1.34	1.09 – 1.65	
	Continuous			1349	1436			0.001
**Total cumulative activity (score)**	1^st^ Tertile	> 0	- ≤ 69	442	446	1.00		
	2^nd^ Tertile	> 69	- ≤ 98	455	496	1.16	0.95 – 1.40	
	3^rd^ Tertile	> 98		452	494	1.19	0.97 – 1.46	
	Continuous			1349	1436			0.11
**Physical workload (score)**	1^st^ Tertile	0	- ≤ 2.656	456	486	1.00		
	2^nd^ Tertile	> 2.656	- ≤ 7.132	448	463	1.06	0.87 – 1.28	
	3^rd^ Tertile	> 7.132		445	487	1.15	0.95 -1.40	
	Continuous			1349	1436			0.13

*Odds ratios (OR) and associated 95% confidence intervals (95% CI) adjusted for Age, Family History and SEIFA Index of Economic Resources.

Table 4 describes the results of the *Polytomous Logistic Regression* comparing the associations between moderate and high grade prostate cancers for each of the exposures. No associations were observed for manual handling of Burdens or for Sedentary Work for either moderate or high grade prostate tumour risk.

**Table 4:** Cumulative occupational exposures for prostate cancer by polytomous logistic regression

**Polytomous logistic regression**										
**Exposure total**	**Exposure**	**Cumulative exposure**		**Moderate grade**			**High grade**			**p –value {heterogeneity**
				**Cases**	OR*	**95% CI**	**Cases**	OR*	**95% CI**	
**Manual handling of burdens (score)**	1^st^ Tertile	0	- ≤ 2.654	446	1.00		88	1.00		
	2^nd^ Tertile	> 2.655	- ≤ 6.808	360	0.93	0.76 – 1.12	83	1.03	0.74 – 1.44	
	3^rd^ Tertile	> 6.808		372	1.01	0.83 – 1.22	87	1.08	0.77 – 1.50	0.81
**Sedentary work (score)**	Unexposed	0		750	1.00		162	1.00		
	1^st^ Tertile	> 0	- ≤ 1.6	153	1.04	0.81 – 1.31	24	0.77	0.48 – 1.22	
	2^nd^ Tertile	>1.6	- ≤ 5.108	132	0.81	0.64 – 1.04	29	0.82	0.54 – 1.26	
	3^rd^ Tertile	>5.108		143	0.98	0.77 – 1.29	43	1.30	0.89 – 1.89	0.21
**Work load (score)**	1^st^ Tertile	> 0	- ≤ 102	312	1.00		54	1.00		
	2^nd^ Tertile	> 103	- ≤ 127	409	1.21	0.99 – 1.48	87	1.36	0.94 – 1.96	
	3^rd^ Tertile	> 128		457	1.35	1.09 – 1.57	117	1.66	1.14 – 2.41	0.57
**Total cumulative activity (score)**	1^st^ Tertile	> 0	- ≤ 69	362	1.00		84	1.00		
	2^nd^ Tertile	> 69	- ≤ 98	422	1.23	1.01 – 1.50	74	0.82	0.58 – 1.16	
	3^rd^ Tertile	> 98		394	1.20	0.98 – 1.46	100	1.05	0.75 – 1.48	0.06
**Physical workload (score)**	1^st^ Tertile	0	- ≤ 2.656	414	1.00		72	1.00		
	2^nd^ Tertile	> 2.656	- ≤ 7.132	360	0.96	0.79 -1.17	103	1.46	1.05 – 2.04	
	3^rd^ Tertile	> 7.132		404	1.14	0.94 -1.38	83	1.22	0.86 - 1.74	0.03

* Odds ratios (OR) and associated 95% confidence intervals (95% CI) adjusted for Age, Family History and SEIFA Index of Economic Resources.

For *total cumulative activity* a significant trend in increasing risk was observed for moderate grade but not high grade tumours (heterogeneity p = 0.06). For *workload*, both moderate and high grade tumours were positively associated with increasing exposure but were not significantly different in this regard. For *physical workload*, a statistically significant trend was observed with increasing levels of exposure but only for high grade tumour risk (heterogeneity p = 0.03).

In this study there were 16,331 reported jobs, providing good variability in job histories for application of FINJEM [21]. The occupational exposure OR’s across all of the variables did not vary substantially from the adjustment models for age, family history and SEIFA Index of Economic Resources in both the binary and polytomous logistic regressions.

## Discussion

This study has found that *workload* is modestly associated with an increased risk of prostate cancer and for both moderate and high grade tumours. This is at odds with the findings of Heikkilä et al. [14] that work related psychological stress is unlikely to be an important factor for prostate cancer. We also found *cumulative activity-over-time* to be modestly associated with prostate cancer risk and showed a small trend with the moderate grade tumours. However, *total cumulative activity-over-time* appears to have a stronger association with the higher grade tumours. These results suggest that the greater the physical activity in the work place over a long period of time the greater the likelihood of the development of high grade prostate cancer.

In comparison with other studies, our finding regarding *total cumulative activity-over-time* is contrary to the findings of Bairati [10] where physical activity in the job had an inverse relationship with prostate cancer and they concluded that physical activity was beneficial. Bairati also found that sedentary/light work had a positive association with prostate cancer whereas our findings showed no associations or trends in regard to prostate cancer. The current analysis also does not support the earlier findings by Ricciardi [4] and Kirk & Rhodes [5] in relation to the combination of OPA and LPTA reduce the risk of advanced prostate cancers. It should be noted that Bairati’s study did not use a Job Exposure Matrix, but instead coded the data related to occupational activity using the five levels of physical activity described by the US Department of Labor.

The major strengths of our study are its sample size and the stratification of the subjects to reflect the population of men with and without prostate cancer. With the three main sites we are confident that the study may be generalizable to the population of men in Australia [21].

In Australia, the treatment of Prostate Cancer is very limited outside state capitals, so our sampling frame is unlikely to be a limitation and has not been compromised due to unrepresentative case ascertainment, even though we recruited cases and controls from three major metropolitan centres of the three selected states. The use of FINJEM in Australia has previously been found to be acceptable for various exposures when compared with expert assessment [18].

The principal weakness of our study is the use of a Job Exposure Matrices (JEMs) that can lead to non-differential misclassification of exposure [22], and we would expect non-differential misclassification to have occurred. This will almost certainly have led to a bias towards null-effect (OR = 1). Multiple hypothesis testing may produce chance positive results, but we did not find anything significant in this regard. The advantage of using a JEM is that it does not rely on self-reported exposure by subjects potentially leading to differential exposure bias from then cases, which is particularly important for more subjective exposure indices such as workload. Being an objective measure of OPA it overcomes the problem of criterion validity of questionnaires [23]. A second limitation is in not having access to BMI’s for the cases and controls, in order to confirm or deny other researchers conclusions [15].

Finally, there would seem to be two contradictory results. Firstly that *manual handling of burdens* which displayed a slight trend with high grade tumours did not show an association with total prostate cancer. However, *Physical workloads* did show a small association with total prostate cancer but no association with either moderate or high grade tumours. Therefore there is insufficient evidence to support any causal relationship and it must be concluded that OPA is unlikely to be beneficial in relation to protecting against PCa, even though other studies have suggested that the combination of OPA and LTPA strongly reduced risk [11,12].

## Conclusions

Our findings are in line with other authors recommendations that suggest further research might be merited in regard to workload and physical workload and prostate cancer risk. We recognize, however, that our findings may point to another more definable psychological agent related to stress in the workplace. Given the modest nature of the associations we describe, we provide little evidence to support any causal relationship and conclude that OPA is not proven to be beneficial in relation to protecting against prostate cancer.

### Consent

A written informed consent was obtained from all participating participants in the original study and this further analysis was undertaken on de-identified data.

## Competing interests

The authors declare that they have no conflict of interests.

## Authors’ contributions

GD participated in the design of this investigation, the acquisition of journal articles, analysis and interpretation of data, the drafting of the manuscript and giving the final approval of the version to be published. GB participated in the design of this investigation, in revising the manuscript critically and for important intellectual content, and giving final approval of the version to be published. GG participated in the design of this investigation, the critical revision of the manuscript for important intellectual content, the drafting of the manuscript and giving final approval of the version to be published. GS consulted on the analysis and interpretation of data and giving final approval of the version to be published. TK participated in the design of this investigation, in revising the manuscript critically and for important intellectual content, and giving final approval of the version to be published. All authors read and approved the final manuscript.
